# Generation of mesenchymal stromal cells from urine-derived iPSCs of pediatric brain tumor patients

**DOI:** 10.3389/fimmu.2023.1022676

**Published:** 2023-01-26

**Authors:** Carmen Baliña-Sánchez, Yolanda Aguilera, Norma Adán, Jesús María Sierra-Párraga, Laura Olmedo-Moreno, Concepción Panadero-Morón, Rosa Cabello-Laureano, Catalina Márquez-Vega, Alejandro Martín-Montalvo, Vivian Capilla-González

**Affiliations:** ^1^ Department of Regeneration and Cell Therapy, Andalusian Molecular Biology and Regenerative Medicine Centre (CABIMER)-CSIC-US-UPO, Seville, Spain; ^2^ Pediatric Surgery Service, Hospital Virgen del Rocio, Seville, Spain; ^3^ Pediatric Oncology Service, Hospital Virgen del Rocio, Seville, Spain; ^4^ Centro de Investigación Biomédica en Red de Diabetes y Enfermedades Metabólicas Asociadas (CIBERDEM), Madrid, Spain

**Keywords:** iPSC, mesenchymal stem/stromal cells (MSC), central nervous system cancer, children, cell therapy, cell reprogramming, oncology, cancer

## Abstract

Human induced pluripotent stem cells (iPSCs) provide a virtually inexhaustible source of starting material for next generation cell therapies, offering new opportunities for regenerative medicine. Among different cell sources for the generation of iPSCs, urine cells are clinically relevant since these cells can be repeatedly obtained by non-invasive methods from patients of any age and health condition. These attributes encourage patients to participate in preclinical and clinical research. In particular, the use of urine-derived iPSC products is a convenient strategy for children with brain tumors, which are medically fragile patients. Here, we investigate the feasibility of using urine samples as a source of somatic cells to generate iPSC lines from pediatric patients with brain tumors (BT-iPSC). Urinary epithelial cells were isolated and reprogrammed using non-integrative Sendai virus vectors harboring the Yamanaka factors *KLF4, OCT3/4, SOX2* and *C-MYC*. After reprogramming, BT-iPSC lines were subject to quality assessment and were compared to iPSCs obtained from urine samples of non-tumor pediatric patients (nonT-iPSC). We demonstrated that iPSCs can be successfully derived from a small volume of urine obtained from pediatric patients. Importantly, we showed that BT-iPSCs are equivalent to nonT-iPSCs in terms of morphology, pluripotency, and differentiation capacity into the three germ layers. In addition, both BT-iPSCs and nonT-iPSCs efficiently differentiated into functional mesenchymal stem/stromal cells (iMSC) with immunomodulatory properties. Therefore, this study provides an attractive approach to non-invasively generate personalized iMSC products intended for the treatment of children with brain tumors.

## Introduction

In 2006, there was a breakthrough in the field of regenerative medicine, when Takahashi and Yamanaka developed the technology to transform any somatic cell into a pluripotent stem cell. These reprogrammed cells, called induced Pluripotent Stem Cells (iPSCs), can be generated by ectopic expression of four transcription factors (i.e. OSKM factors): octamer binding transcription factor 3/4 (*OCT3/4*), sex determining region Y-box 2 (*SOX2*), Krüppel-line factor 4 (*KLF4*) and cellular-myelocytomatosis (*C-MYC*) (1, 2). Similar to embryonic stem cells, iPSCs have the ability to self-renew and differentiate into any specialized cell of the body. In addition, iPSCs have certain advantages over other stem cell types for cell-based therapies. Firstly, iPSCs avoid the ethical concerns about the use of embryos to generate pluripotent stem cells. Secondly, they provide a virtually unlimited supply of human cells, bringing the possibility of generating personalized cells for autologous treatment, preventing immune rejection. Importantly, iPSC technology allows the manufacturing of next generation cells, such as iPSC-derived mesenchymal stem/stromal cells (iMSCs), which have been shown to have increased therapeutic efficacy when compared to tissue-derived mesenchymal stem/stromal cells (MSCs) in pre-clinical studies (3–8). Thirdly, these cells can be harvested from easily accessible sources, such as skin, blood or urine. Particularly, urine-derived iPSCs are obtained by simple, pain-free methods, reducing the risk of adverse effects associated with invasive collection procedures. Therefore, urine offers an interesting option to collect cells repeatedly from patients of any age and under any medical condition, such as pediatric cancer patients (9–11).

Brain tumors are the most common solid tumor in children and represent the leading cause of pediatric cancer-related deaths. Latest advances in diagnosis and treatments have improved survival rates of children suffering brain tumors. However, adverse effects of cancer therapies are still affecting the health of many brain tumor survivors. For this reason, researchers are focusing on the development of new strategies aimed to reduce toxicity of cancer treatments. The majority of brain tumor patients that receive radiotherapy, one of the most common treatments for cancer (12), exhibit cognitive dysfunction, including deficits in learning, memory, language, attention and executive function (13). These neurological complications are frequently associated with radiation-induced damage to healthy brain tissue, such as neuroinflammation and cell death (12, 14–17). Despite the fact that neurocognitive sequelae of radiotherapy may occur in patients of any age, these adverse effects particularly affect pediatric patients because the developing brain is more sensitive to radiation.

Recent reports have demonstrated the neuroprotective effects of cell-based therapies to prevent neurological complications of radiotherapy, thus promoting a healthy cancer-free life (18–24). In particular, the administration of MSCs has been shown to efficiently rescue behavioral deficits in mice following cranial radiation, by reducing neuroinflammation and cell death (18, 22–24). However, the clinical translation of MSC-based therapies as a neuroprotective strategy is hampered by challenges related to manufacturing and cell availability. In this context, the use of iPSCs as an unlimited source of MSCs (i.e., iMSCs) emerges as an interesting option that enables large-scale production of cellular products for both autologous and allogenic therapies. Furthermore, iMSCs can generate from a single iPSC clone, thus reducing the heterogeneity acquired by tissue-derived MSCs (25–27). These attributes facilitate the obtaining of consistent and robust final products that could be used for the prevention of radiation-related neurological complications. However, the generation of iMSCs from brain tumor pediatric patients remains to be achieved.

In this study, we demonstrated that urine-derived epithelial cells (UDCs) is a feasible source to generate iPSCs from children with brain tumors following a non-invasive cell collection procedure. In addition, we show that iPSCs differentiate into functional iMSCs. To our knowledge, this is the first study involving brain tumor pediatric patients that successfully generate iMSCs. The establishment of iPSC lines offers a stable source of MSCs (i.e., iMSCs), boosting their clinical application in a variety of diseases, for both children and adults.

## Methods

### Obtaining of urine samples from pediatric patients

**Figure 1:**
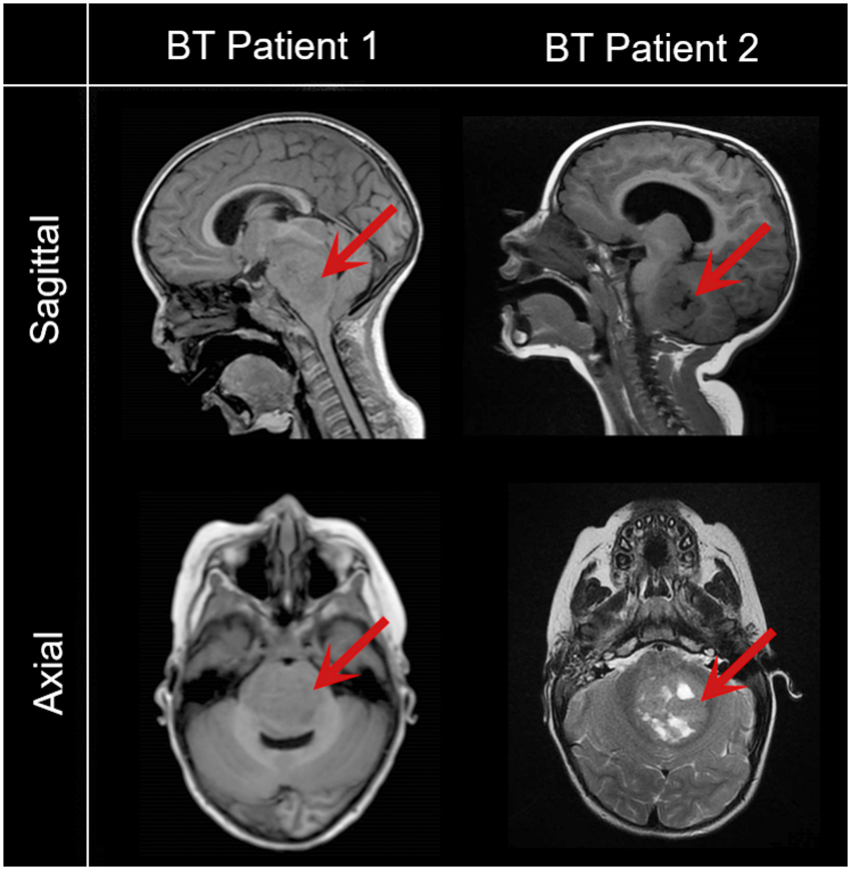
Representative magnetic resonance images of pediatric patients with brain tumors included in the study. Saggital and axial images reveal a low grade glioma (BT Patient 1, 43-month-old) and a metastatic brain tumor (BT Patient 2, 24-month-old). Red arrows point to the tumor.

Urine samples were collected from pediatric patients (age <60 months old) at the Hospital Universitario Virgen del Rocío of Seville, after obtaining written informed consent. Samples with volumes ranging from 12 to 40 ml were collected in a sterile container, kept at 4° C, and processed within 1 hour. Urine obtained from brain tumor patients was collected prior to oncological treatment (e.g. surgery, radiotherapy or chemotherapy). Urine obtained from non-oncological patients was used as control samples (**Table 1**, **Figure 1**).

**Table 1:** Clinical characteristics of pediatric patients.

Patient	Age (months)	Sex	Diagnosis	Sample Volume (urine)	hPSCreg name	Sample ID*
nonT Patient 1	7	male	Inguinal hernia	12.6 mL	ESi089-A	nonT-iPSCs 1
nonT Patient 2	56	male	Cryptorchidism	13.6 mL	ESi090-A	nonT-iPSCs 2
BT Patient 1	43	female	Low grade Glioma	32 mL	ESi087-A	BT-iPSCs 1
BT Patient 2	24	female	Metastatic brain tumor	40 mL	ESi088-A	BT-iPSCs 2

* Sample ID relates to the name given to each iPSC line in this manuscript.

### Isolation and expansion of UDCs

Urine was centrifuged at 400 × g for 10 min at room temperature. Cell pellets were washed with Dulbecco’s phosphate buffered saline (DPBS), resuspended in isolation medium (DMEM/F-12 with 15mM HEPES, 10% fetal bovine serum (FBS), 1% non-essential amino acids, 10 ng/mL recombinant human EFG, 36 ng/mL hydrocortisone, 5 µg/mL recombinant human insulin, 500 ng/mL epinephrine, 5 µg/mL human holo-transferrin, 4 pg/mL triodo-L-thyronine, 434.4 µg/mL alanyl-glutamine, 100 µg/mL penicillin/streptomycin, 2.5 µg/mL amphotericin B and 0.1% rock inhibitor) and seeded in gelatin-coated plates. Half-medium changes were performed every day to avoid unnecessary cellular stress. Once the first colonies were observed (day 7-15), total medium was replaced. The first passage was performed when cells were approximately 30% confluent using trypsin (day 12-20). Then, UDCs were seeded at a density of 10.000 cells/cm^2^ with a 1:1 mix of isolation medium and expansion media (i.e., isolation medium with reduced FBS concentration to 5% and antibiotic free). From passage 2, only expansion medium was used to boost UDC amplification prior to cell reprograming. UDCs were incubated at 37°C in a humidified atmosphere with 20% O_2_ and 5% CO_2_.

### Reprogramming of UDCs into iPSCs

**Figure 2:**
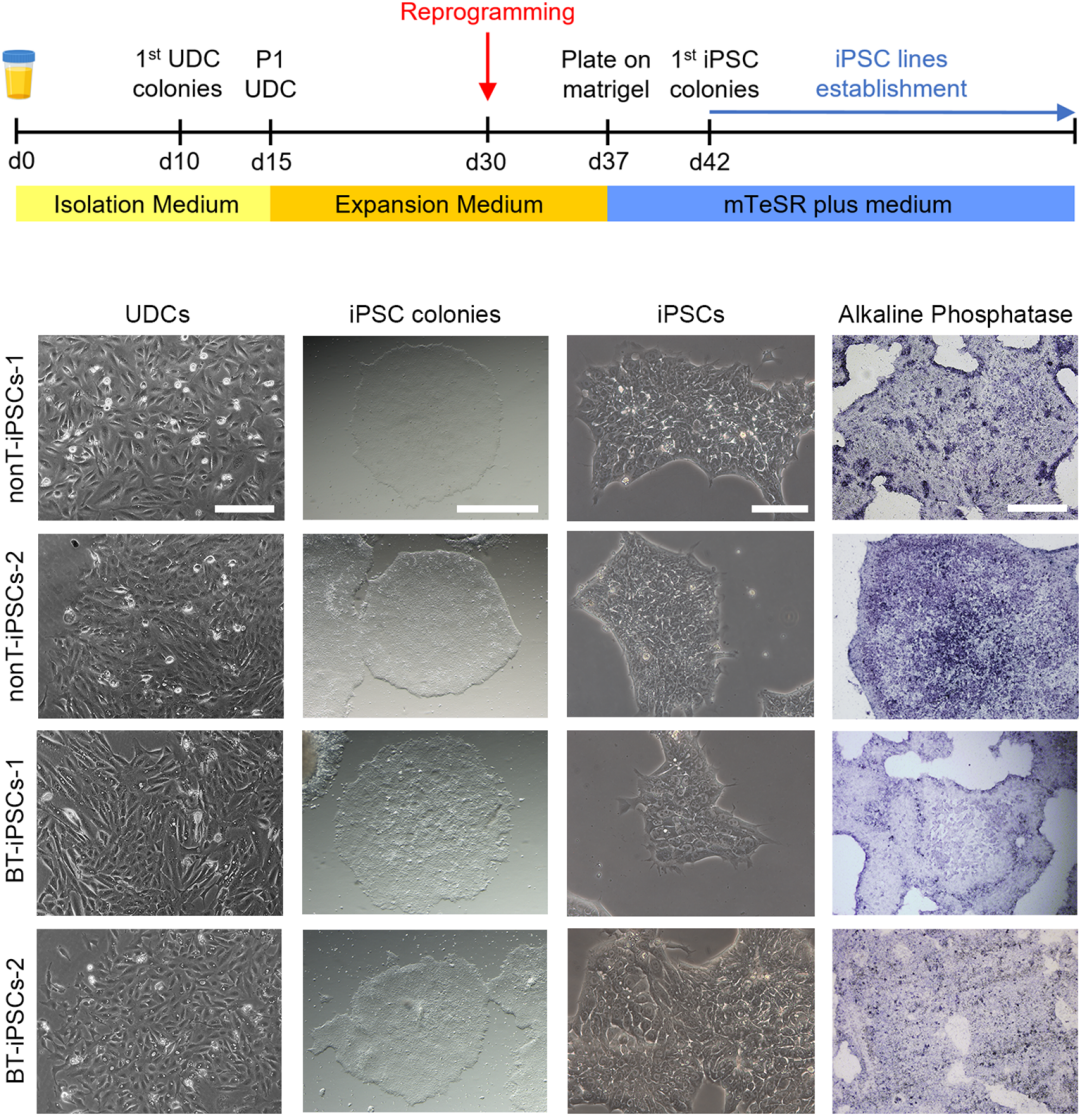
Isolation of UDCs and reprogramming process. **(A)** Schematic timeline of the process for isolation and expansion of UDCs and iPSC generation. **(B)** Microscope images showing the morphological aspect of cultured UDCs (scale bar 200 µm), iPSC colonies obtained after selective passaging post-reprogramming (scale bar 1 mm), iPSCs at higher magnifications (scale bar 100 µm) and alkaline phosphatase staining of iPSC colonies (scale bar 400 µm).

UDCs at passage below 5 were reprogrammed when cells reached a 30-60% confluency. To reduce the risk of genetic abnormalities, UDCs were reprogrammed to iPSCs using the non-integrative CytoTune™-iPS 2.0 Sendai Reprogramming Kit (Thermo Fisher, Waltham, MA, USA) (**Figure 2A**). After a 7-day period, transfected cells were transferred onto a matrigel-coated plate with mTeSR plus basal medium (STEMCELL Technologies, Grenoble, France) and allowed to grow in a humidified incubator with 37°C, 20% O_2_ and 5% CO_2_. After 5 days, iPSC colonies emerged. The first 4 passages were carried out mechanically to specifically select colonies with an iPSC morphology. Then, cell passages were performed using the ReLeSR reagent (STEMCELL Technologies), following the manufacturer’s guidelines. iPSCs were expanded for full characterization and banking. All established cell lines will be deposited at the Banco Nacional de Líneas Celulares (BNLC) of the Institute of Health Carlos III, following the Spanish legislation.

### Characterization of the generated iPSCs

#### Alkaline phosphatase staining

Alkaline phosphatase activity was evaluated in fixed iPSC colonies using the SIGMAFAST™ BCIP^®^/NBT kit (Sigma-Aldrich, St. Louis, MO, USA), following the manufacturer’s guidelines. Stained iPSCs were visualized and imaged using an Olympus IX71 microscope equipped with an DPController and DPManager software (Center Valley, PA, USA).

#### Three lineage differentiation

For the differentiation of iPSCs into the three germ layers (endoderm, mesoderm and ectoderm), we used the specific induction media of the STEMdiff™ Trilineage Differentiation Kit (STEMCELL Technologies). According to the manufacturer’s instructions, a 5-day period was required for endoderm and mesoderm differentiation, while ectoderm induction needed 7 days. Media was changed daily. Differentiated cells were harvested and used to isolate RNA for reverse transcriptase‐polymerase chain reaction (RT-PCR) and to perform immunofluorescence assays.

#### Immunofluorescence staining

Cells were fixed with 4% paraformaldehyde (PFA), washed with phosphate buffered saline (PBS), incubated in blocking solution for 1 hour, and incubated with primary antibodies at 4°C overnight (see **Supplemental Table 2** for antibodies information). Then, cells were washed and incubated with the appropriate secondary antibodies conjugated with fluorophores. Hoechst 33342 nucleic acid stain (Sigma-Aldrich) was used to detect cell nuclei. Fluorescence labeling was examined using an Olympus IX71 microscope equipped with DPController and DPManager software.

#### Molecular karyotyping

DNA from iPSCs was extracted using the DNeasy Blood and Tissue kit (QIAGEN, Gilden, Germany). Then, molecular karyotyping was performed with an Affymetrix CytoScan 750k Array (Affymetrix, Santa Clara, CA, USA) by trained experts of the Genome Core Facility of CABIMER to detect chromosomal variations (log2 ratio intensities and copy number). Data viewing and analysis was performed with the Affymetrix Chromosome Analysis Suite (ChAS) software, using the standard setting filters (400 kbp with a marker count of ≥50 for gains; 400 kbp with a marker count of ≥25 for losses) and compared to control data from the Database of Genomic Variants (DGV). The Online Mendelian Inheritance in Man (OMIM) database was used to study associations of genetic alterations with disease susceptibility. All samples and processes fulfilled the following quality criteria: MAPD ≤ 0.25, Waviness SD ≤ 0.12 and SNPQC ≥15. Affymetrix data are deposited in Gene Expression Omnibus (GEO) database repository (accession number: GSE213813).

#### Fingerprinting

The identity of the established cell lines was analyzed by the cell line authentication service qGenomics (Barcelona, Spain). DNA analysis of the iPSCs and their parental UDCs was performed by genotyping the following human-specific short tandem repeat (STR) markers: *TH01*, *D21S11*, *D5S828*, *D13S317*, *D7S820*, *D16S539*, *CSF1PO*, *vWA* and *TPOX*. The combination of these nine genetic markers results in an allele profile with a random match probability of 1 in 2.9 x 10^9^. A gender-specific marker (*AMEL*) was also analyzed to establish the presence of sex chromosomes, as well as a specific marker to detect the presence of murine sequences that could result from a possible cross-contamination of the cultured iPSCs with any independent culture of murine cells.

### Differentiation of iPSCs into MSCs and characterization

Differentiation of iPSCs into iMSCs was induced with the STEMdiff™ Mesenchymal Progenitor Kit (STEMCELL Technologies), following the manufacturer’s instructions. Briefly, iPSCs were differentiated into early mesoderm progenitor cells for four days. Then, cells were plated in animal component-free (ACF) precoated wells to derive early mesenchymal progenitor cells. By day 21, differentiated cells exhibited MSC phenotype that was verified by morphological analysis, flow cytometry and multilineage differentiation potential, based on previously published methodology (28, 29). For the flow cytometry study, the surface markers CD13, CD14, CD73, CD105, CD29, CD31, CD34, CD45, CD90 and HLA II were used (see **Supplemental Table 2** for antibody information). Differentiation of iMSCs into adipocytes was performed using the MesenCult™ Adipogenic Differentiation Kit (STEMCELL technologies). After 21 days, the presence of lipid droplets was determined by staining the cells with Oil red O (Sigma-Aldrich) after fixation with 4% PFA for 5 minutes. Differentiation of iMSCs into osteoblasts was performed using the MesenCult™ Osteogenic Differentiation Kit (STEMCELL technologies). After 15 days, the presence of osteoblasts was determined by staining the cells with Alizarin Red S sodium salt (Alfa Aesar, Haverhill, MA, USA), after fixation with 4% PFA for 5 minutes. Differentiation of iMSCs into chondrocytes was performed using the MesenCult™-ACF Chondrogenic Differentiation kit (STEMCELL technologies). After 21 days, cell pellets were fixed overnight with 4% PFA, paraffin-embedded and sectioned with a microtome (Leica RM 2255) to 5 µm thickness. Then, sections were stained with Alcian-Blue solution (Sigma-Aldrich). Images were visualized with an Olympus IX71 microscope and a Nikon ECLIPSE Si microscope.

### Inflammatory cytokines secreted by iMSCs

Culture medium was collected after 48 hours of cell growth to analyze cytokines secreted by iMSCs. Interleukin 8 (IL-8), interleukin 12p70 (IL-12p70), monocyte chemoattractant protein-1 (MCP-1, also named CCL2), platelet-derived growth factor BB (PDGF-BB) and tumor necrosis factor alpha (TNFα) were examined using the Quantibody Human Inflammation Array-3 (Raybiotech, Inc., Norcross, GA, USA), according to the manufacturer´s protocol. Fluorescence signals were detected at 532 nm by a laser scanner (Axon GenePix; Molecular Devices, Sunny-vale, CA, USA).

### iMSC priming

Cells were incubated during 48 hours with (primed iMSC) or without (non-primed iMSC) an inflammatory cytokine mixture containing recombinant human TNFα (10 ng/mL) and recombinant human Interferon gamma (IFNγ; 10 ng/mL) in DMEM supplemented with 15% FBS. For gene expression analysis, a set of cells was thoroughly washed after priming to remove these cytokines and collected for RNA isolation either inmediatly or 48 hours after priming. For secretome generation, another set of cells was thoroughly washed after priming and then fresh culture medium was added. After a 48 hours period, conditioned medium (CM) was collected and stored at -80° until use.

### RNA extraction and RT-PCR

Total RNA was isolated from cultured cells with the Easy-Blue™ kit (iNtRON Biotechnology, Inc., Seongnam, Korea). cDNA was obtained from 1 μg of total RNA using the iScript cDNA synthesis kit (Bio-Rad Laboratories, Hercules, CA, USA). Conventional RT-PCR was used to evaluate the expression of pluripotency- and differentiation-associated genes using the primers described in **Supplemental Table 1**. The clearance of the Sendai virus (SeV) vector and the OSKM reprogramming factors was also confirmed by conventional RT-PCR. Agarose gel electrophoresis was used to resolve PCR products. Presence of mycoplasma was analyzed using the commercial kit Venor GeM (Minerva Biolabs GmbH, Berlin, Germany). For quantitative RT-PCR (RT-qPCR), the iTaq Universal SYBR Green Supermix (Bio-Rad Laboratories) was used following the manufacturer guidelines, using a total volume of 10 µL and 50 ng of cDNA per reaction, in triplicates. RT-qPCR was performed using a ViiA™ 7 Real-Time PCR System (Applied Biosystems, Foster City, CA, USA) and analyzed with the ViiA™ 7 Software (Applied Biosystems), using the standard instrument protocol. The expression of immunomodulatory genes was studied using the primers described in **Supplemental Table 1** and the relative gene expression was normalized using TATA box binding protein (TBP) as the housekeeping gene. Data was collected from three independent experiments.

### iMSC and PBMC cocultures

Peripheral blood mononuclear cells (PBMCs) from two donors with different blood types were purchased from STEMCELL Technologies (Cat# 70025.2). PBMCs were thawed and cultured overnight in RPMI medium supplemnted with 10% FBS, 1% penicillin/streptomycin, and 1% L-glutamine to allow resumption of metabolism, as suggested by the manufacturer. ImmunoCult™ Human CD3/CD28 T Cell Activator reagent (Cat# 10971, STEMCELL Technologies) was used to stimulate PBMCs, following the manufacturer’s instructions (25 µl of reagent per ml of medium). For direct cocultures, 1·10^5^ activated PBMCs were added to cultured iMSCs at different ratios (PBMC:iMSC of 2:1, 4:1, 8:1, 16:1) in a 96 well plate for 5 days (30). For indirect cocultures, 1·10^5^ activated PBMCs were incubated for 72h either in RPMI or in a combination of RPMI and CM from primed/non-primed iMSC (volume ration RPMI : RPMI:CM of 1:1) in a 96 well plate. The CM was generated from 1·10^6^ iMSCs cultured for 2 days in 10 ml of medium. To study the proliferation of CD3+ T cell, PBMCs were labelled with the cell division tracker 5-chloromethylfluorescein diacetate (C7025, Thermo Fisher) and then subjected to flow cytometry (31). To evaluate the proliferation of CD4+ and CD8+ T cell subpopulations, a Ki-67 proliferation test was performed following the manufacturer´s protocol (32).

### Flow cytometry

Cultured cells were harvested and incubated for 20 minutes with the appropriated primary antibodies in the dark at room temperature (see **Supplemental Table 2** for antibody information). For Ki-67 staining, cells were fixed and permeabilized with ethanol at -20° C for 2 hours before incubation with primary antibody. After primary antibody incubation, cells were washed with PBS, centrifuged at 2000 rpm for 5 minutes and analyzed using an LSRFortessa X-20 flow cytometer (BD Biosciences, San Diego, CA, USA) and the BD FACSDiva software (BD Biosciences).

### Determination of superoxide dismutase activity

The antioxidant action of the iMSC secretome was measured by the Superoxide Dismutase (SOD) Activity Assay Kit (Cayman Chemical, Ann Arbor, MI, USA). Samples were assayed according to the manufacturer’s instructions. Absorbance was measured at 450 nm using a Varioskan Flash microplate reader (Thermo Electron, Vantaa, Finland). Units of SOD activity were calculated from a standard curve using purified bovine erythrocyte SOD enzyme. All measurements were performed in duplicate.

### Statistical analysis

Data expressed as mean ± SEM was analyzed using the GraphPad Prism 8 software (GraphPad Software Inc., San Diego, CA). Parametric ANOVA followed by a Tukey´s or Dunnett’s *post-hoc* test was performed to compare more than two experimental groups. Comparison between two experimental groups were performed with Student’s t-test. All differences were considered significant at a P value <0.05.

## Results

### UDCs from children with brain tumors can be successfully cultured and reprogrammed into iPSCs

The isolation of epithelial cells was carried out from urine samples of four pediatric patients, two with brain tumors (BT patients) and two with non-tumor conditions (nonT patients) (**Figure 1**, **Table 1**). Adherent UDCs were observed during the first week of culture, showing spindle-shaped or round-shaped morphologies. First UDC colonies emerged on day 11 and, then, UDCs were expanded for a maximum of 5 passages to assure enough material for reprogramming (**Figure 2**).

To generate iPSCs, we used the non-integrative Sendai viral vectors to reduce the risk of genetic abnormalities in the generated cell lines (**Figure 2A**). The reprogramming efficiency was similar in all patient-derived samples. At day 7 post-reprogramming, colonies with typical iPSC morphology (i.e. dense, roundly shaped colonies with sharp edges, containing small cells with a high nucleus-to-cytoplasm ratio) were selected and manually picked to achieve highest culture purity. Alkaline phosphatase activity staining confirmed the identification of pluripotent iPSC colonies (**Figure 2B**). Passaged iPSCs were seeded onto matrigel-coated plates and expanded for full characterization.

**Figure 3:**
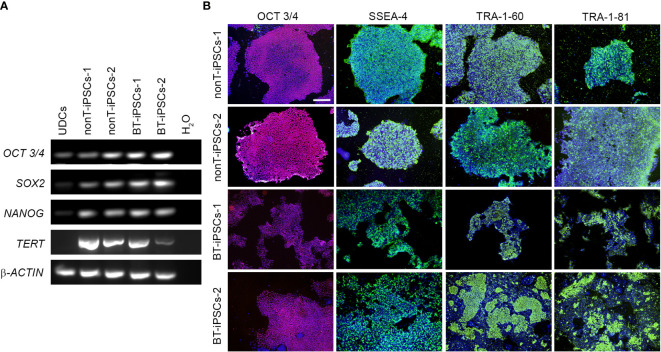
Analysis of the pluripotency markers in the generated iPSC lines. **(A)** RT-PCR analysis of the expression of pluripotency-associated genes. Note that all iPSC lines highly express *OCT3/4, SOX2, NANOG* and *TERT*, while UDCs do not. **(B)** Immunofluorescence staining showing the presence of pluripotency markers OCT3/4 (magenta), SSEA-4 (green), TRA-1-60 (green) and TRA-1-81 (green) in the iPSCs. Nuclei were counterstained with Hoechst 33342 (blue). Scale bar 100 µm.

To further validate the identity of the generated cell lines, gene expression of pluripotency markers was evaluated. RT-PCR analysis evidences that, in contrast to UDCs, iPSC colonies from BT patients and nonT patients had a robust expression of the key pluripotency genes *OCT3/4, SOX2, NANOG* and *TERT* (**Figure 3A**). We also evaluated the expression of pluripotency markers at protein level by immunofluorescence, using the intracellular marker OCT3/4 and the surface markers SSEA4, TRA-1-60 and TRA-1-81. Cultured iPSCs stained positive for all the markers assessed, confirming their pluripotency (**Figure 3B**).

### Multilineage differentiation potential was confirmed in patient-derived iPSCs

**Figure 4:**
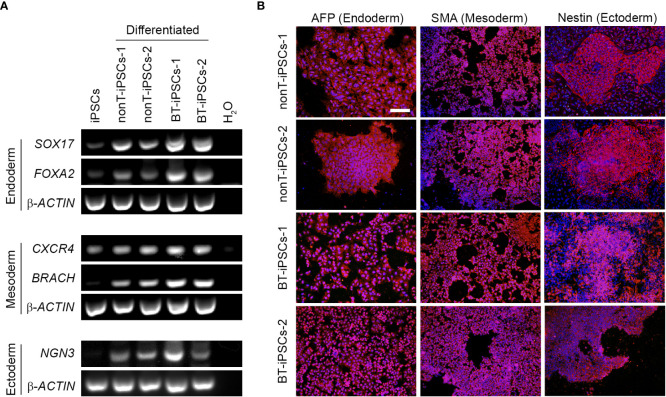
Analysis of the differentiation capacity of the generated iPSCs into the three embryonic layers. **(A)** RT-PCR analysis showing the expression of specific differentiation markers, including markers for endoderm (*SOX17* and *FOXA2*), mesoderm (*CXCR4* and *BRACH*) and ectoderm (*NGN3*). Note that differentiated iPSCs highly express all the trilineage differentiation markers, while undifferentiated iPSCs do not. **(B)** Immunofluorescence staining showing the presence of alfa-fetoprotein (AFP) for endoderm, Smooth Muscle Actin (SMA) for mesoderm and Nestin for ectoderm in the differentiated iPSCs. Nuclei were counterstained with Hoechst 33342. Scale bar 50 µm.

To further demonstrate the pluripotency of iPSCs, tri-lineage differentiation was performed using specific induction media. Reprogrammed cells efficiently differentiated into endodermal, mesodermal and ectodermal lineage cells. The differentiation potential of the generated iPSCs was confirmed through expression of specific genes for endoderm (*SOX17, FOXA2*), mesoderm (*CXCR4, BRACH*) and ectoderm (*NGN3*) (**Figure 4A**). In addition, differentiated iPSCs were assessed for immunofluorescence using AFP (endoderm marker), SMA (mesoderm marker) and Nestin (ectoderm marker) (**Figure 4B**). Both RT-PCR and immunofluorescence results denoted similar tri-lineage differentiation potential in all patient-derived iPSC lines.

### Authentication, molecular karyotyping, virus clearance and mycoplasma testing of established iPSC

**Figure 5:**
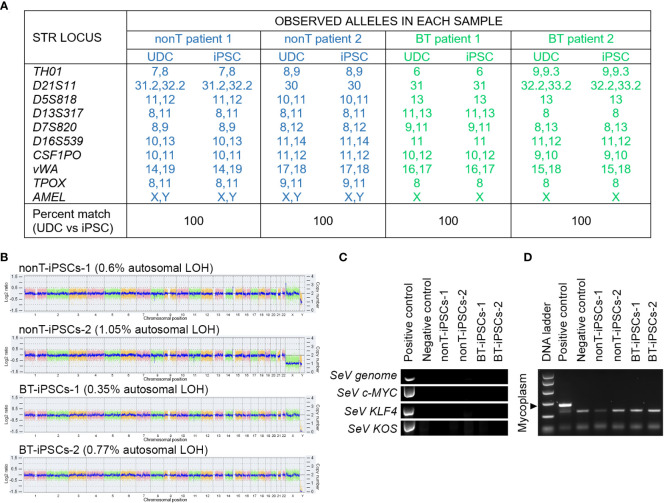
Authentication, molecular karyotyping, virus clearance and mycoplasma testing of established iPSCs. **(A)** DNA fingerprint analysis showing that the allele pattern in the iPSCs generated is 100% concordant with the patients’ UDCs and it is not concordant with any commercial cell line whose genotype is posted in public databases. The STR locations studied were: *TH01, D21S11, D5S818, D13S317, D7S820, D16S539, CSF1PO, vWA, TPOX* and *AMEL*. The percentage of matching between iPSCs and their parental UDCs is indicated for each sample. **(B)** Whole genome view of the iPSC lines which displays all somatic and sex chromosomes in one frame. The smooth signal plot (right y-axis) is the smoothing of the Log2 ratios (left y-axis), which depict the signal intensities of probes on the microarray and represents the number of copies of each chromosome. The pink, green and yellow colors represent the raw signal for each individual chromosome probe, and the blue signal represents the normalized probe signal, used to identify copy number and any aberrations. The percentage of autosomal loss of heterozygosity (LOH) is indicated for each cell line. **(C)** RT-PCR analysis showing the absence of expression of Sendai virus (SeV) genome and OSKM transgenes in the established iPSC lines. UDCs served as negative control and recently transfected iPSCs served as positive control. **(D)** PCR test for mycoplasma detection, showing the absence of contamination in cultured iPSC lines.

DNA fingerprinting analysis indicates that iPSCs from BT and nonT patients shared identity with their parental UDCs, demonstrating that we generated four new iPSC lines (**Figure 5A**). The safety of the reprogramming method was evaluated by molecular karyotyping, which evidences minimal chromosomal abnormalities (<1.1% for autosomes) in all urine-derived iPSCs, based on Log2 ratio and copy number (**Figure 5B**). Importantly, genomic alterations related to tumorigenesis were absent in the iPSCs from brain tumor pediatric patients (**Supplemental Table 3**). Finally, the absence of exogenous reprogramming vectors and mycoplasma contamination was verified in the generated iPSC lines by negative PCR (**Figures 5C, D**). These results support that the production of iPSCs from pediatric patients with brain tumors may be suitable for clinical applications.

### Differentiation of iPSCs into iMSCs

**Figure 6:**
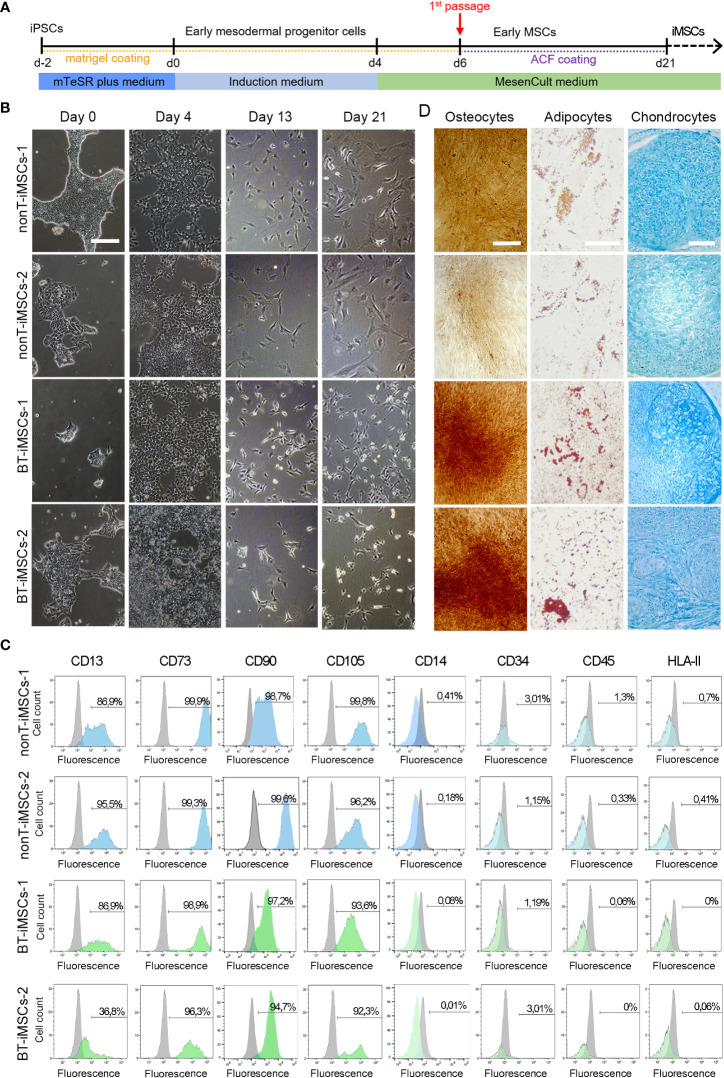
Directed differentiation of iPSCs towards iMSCs. **(A)** Schematic timeline of the process for differentiation into iMSCs. **(B)** Microscope images showing the morphological aspect of differentiated iMSCs over time (scale bar 100 µm). **(C)** Flow cytometry analysis of differentiated iMSCs showing that cells were positive for the MSC-specific markers CD13, CD73, CD90 and CD105, whereas they were negative for CD14, CD34, CD45, and HLA-II. **(D)** Representative images of the differentiated iMSCs into osteocytes (scale bar 100µm), adipocytes (scale bar 50µm) and chondrocytes (scale bar 100µm) identified by Alizarin Red, Oil Red O and Alcian Blue staining, respectively.

In order to determine whether iPSCs can serve as a platform to obtain next generation MSCs, we used specific induction media to generate iMSCs (**Figure 6A**). During the course of the protocol, iPSCs from BT and nonT patients acquired a fibroblast-like morphology (**Figure 6B**). By day 21, differentiated iPSCs expressed typical MSC markers, such as CD13, CD29, CD73, CD90 and CD105, while they lacked the expression of CD14, CD31, CD34, CD45, and HLA-II (**Figure 6C**, **Supplemental Figure 1**). In addition, the absence of undifferentiated cells (i.e. iPSCs) was confirmed by the lack of expression of TRA-1-60 and SSEA4, supporting successful differentiation of iPSCs into MSCs (**Supplemental Figure 2**). iMSC identity was further confirmed based on their multilineage differentiation potential. Both BT and nonT patient-derived iMSC possessed the ability to differentiate into adipocytes, osteocytes and chondrocytes, as demonstrated by the positive staining for Oil Red O, Alizarin Red S and Alcian Blue, respectively (**Figure 6D**, **Supplementary Figure 1**). These data demonstrate that urine-derived iPSCs from pediatric patients can be efficiently differentiated into iMSCs, irrespective of brain tumor diagnosis.

### Immunomodulatory potential of the generated iMSCs

**Figure 7:**
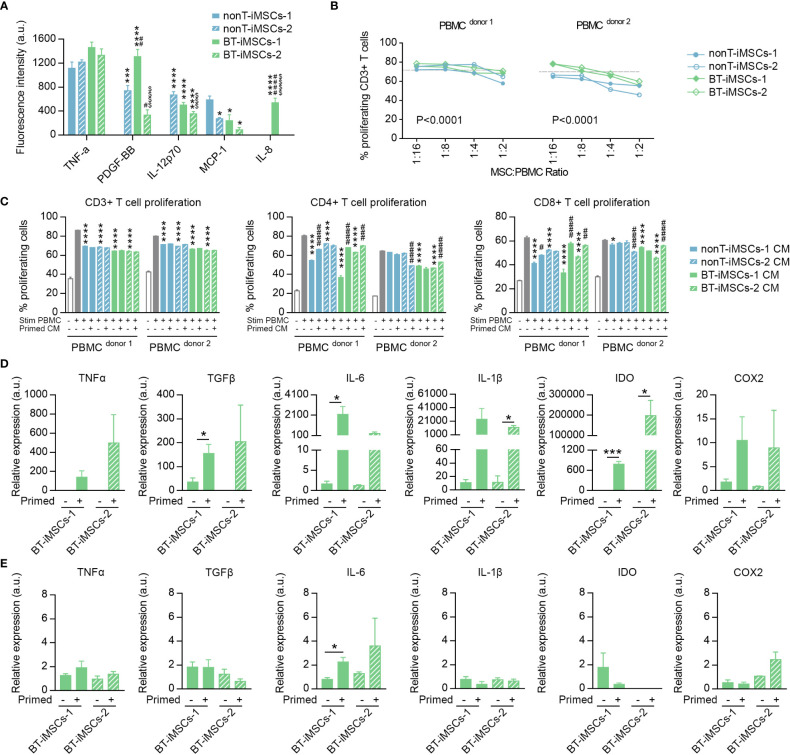
Immunomodulatory potential of the generated iMSCs. **(A)** Bar graph showing the fluorescence intensity for inflammatory cytokines secreted by iMSCs determined by ELISA. **(B)** Inhibitory effect of iMSCs on CD3+ T cell proliferation after 5 days co-culture. Dashed line indicates the % of proliferation for CD3+ T cells without iMSCs. **(C)** Bar graphs showing the percentage of CD3^+^, CD4^+^ and CD8^+^ proliferating T cells in response to the secretome of primed or non-primed iMSCs. Of note, the secretome was collected 48 hours after priming. **(D)** Bar graphs showing the expression levels of immunomodulatory genes analyzed by RT-qPCR in primed and non-primed iMSCs derived from BT patients. Of note, the RNA was isolated immediately after priming. **(E)** Bar graphs showing the expression levels of immunomodulatory genes analyzed by RT-qPCR in primed and non-primed iMSCs derived from BT patients. Of note, the RNA was isolated 48 hours after priming. Data are represented as mean ± SEM. The absence of bar in any graph indicates undetected levels of the specific parameters assessed. For **(A)**; *p*0.05, ***p<0.001, ****p<0.0001 compared to nonT-iMSCs-2; ^#^p<0.05, ^##^p<0.01, ^####^p<0.0001 compared to nonT-iMSCs-1; $$$p<0.001, $$$$p<0.0001 compared to BT-iMSCs-1. One-way ANOVA. For **(B)**; P value refers to dose effects. Two-way ANOVA. For **(C)**; *p<0.05, ****p<0.0001 compared to stimulated PBMC cultured in regular growth medium. ^#^p<0.05, ^##^p<0.01, ^###^p<0.001, ^####^p<0.0001 compared to their primed counterparts. One-way ANOVA. For **(D-E)**; *p<0.05, ***p<0.001. Student's t-test.

To evaluate the immunomodulatory phenotype of the generated iMSCs, we examined the secretion of immunoregulatory cytokines in the media of cultured cells. We analyzed the presence of IL-8, IL-12p70, MCP-1, PDGF-BB and TNFα and observed that all the iMSCs secrete at least two of the analyzed cytokines (**Figure 7A**). Then, we assessed the capacity of iMSCs to reduce T cell proliferation using activated PBMCs from different donors, in both direct and indirect cocultures (**Figures 7B, C**). We showed that the generated iMSCs cultured in direct contact with PBMCs inhibited CD3+ T cell proliferation in the PBMC of the two donors tested (**Figure 7B**). In addition, we also determined that the proliferation of CD3+ T cells was diminished by the secretome of BT-iMSC and nonT-iMSC, with an overall similar trend in both CD4+ and CD8+ T cell subsets (**Figure 7C**). Despite the fact that comparison of iMSCs with adipose tissue-derived MSCs (adMSCs) was not the focus of this study, we would like to mention that the secretome of iMSCs exhibited higher capacity to decrease CD3+ T cell proliferation and showed enhanced antioxidant capacity when compared to adMSCs (**Supplemental Figures 3A-C**). Furthermore, the secretome of primed iMSCs was used to interrogate the immunomodulatory plasticity of MSCs after stimulation with inflammatory cytokines (33, 34). We found that cell priming with TNFα and IFNγ modulated the immunomodulatory potential of iMSC secretome, showing in several instances a predisposition to dampen the inhibition of T cell proliferation when compared to the CM of non-primed iMSCs (**Figure 7C**). These results demonstrated that urine-derived iPSCs from BT and nonT patients are a viable source to obtain iMSCs with immunomodulatory properties.

In order to investigate how the priming with inflammatory cytokines regulates BT-iMSC immunomodulation, we examined themRNA levels of several immunomodulatory genes, including TNFa, TGFb, IL-6, IL-1b, IDO and COX2, in BT-iMSCs. While an overall increase of these factors was found immediately after cell priming (**Figure 7D**), the expression of the immunomodulatory genes tested reverted to basal levels after 48 hours of priming, with minimal residual effects on IL-6 expression in primed cells (**Figure 7E**). These data suggest that a transient upregulation of immunomodulatory genes in iMSCs is sufficient to improve their immunomodulatory secretome.

## Discussion

Reprogramming somatic cells into iPSCs has emerged as an innovative strategy to improve manufacturing of cellular products for regenerative medicine. Among the different somatic cell types, UDCs represent a convenient source of iPSCs for personalized cell therapies, particularly for those patients with a severe disability affecting their health. Among medically fragile patients, children with cancer are at a high risk of frailty due to the progression of the disease and to the side effects of cancer treatments. For instance, brain tumor pediatric patients experience multiple neurological complications, including deficits in learning, memory, language, attention and processing speed, even after overcoming the illness (35). These debilitating conditions impede in many cases their participation in clinical trials, which is a major challenge for the translation of pediatric cancer research into clinical practice. Unlike solid biopsies, urine collection represents an easy, safe, pain-free and inexpensive way of obtaining large quantities of somatic cells to engineer cell-based therapeutics for a variety of diseases (11, 36, 37). Here we report for the first time the generation of two iPSC lines from UDCs of children with brain tumors. Our results demonstrated that iPSCs can be obtained from small volumes of urine (12-40 ml range), allowing the generation of patient-specific cellular products for therapeutic use, without the need for invasive biopsies. The use of non-invasive sampling methods could increase the number of patients that can benefit from cell therapies, irrespective of their medical condition. Importantly, we used the non-integrative Sendai viral vectors, thus reducing the risk of generating genetic alterations. All these important aspects represent a decisive boost for personalized medicine in the field of childhood cancer. The application of an iPSC-based therapy using autologous cells will prevent an immune response, as the host will recognize the transplanted cell products as their own. In a recent study, personalized iPSC-derived dopamine progenitor cells were successfully applied in a patient with Parkinson’s disease, who exhibited clinical improvements and survived without the need for immunosuppression (38). This evidences that, in contrast to allogeneic cell products, patient-specific cells may be safe in terms of immunoreaction. Therefore, the use of iPSC technology might represent a feasible manner to generate safe and cost-effective cell-based products.

All patient-derived UDCs in this study were successfully reprogrammed into iPSCs, which underwent exhaustive characterization by assessing their pluripotency and multilineage differentiation potential. In addition, we tested iPSC identity, karyotype, virus clearance and mycoplasma contamination, basic quality attributes that are required for cell banking. The storage of high-quality iPSCs allows for clinical application under autologous and allogenic conditions, but also provides researchers the opportunity to conduct iPSC-based studies when they are unable to generate iPSCs in their own labs. In addition to cell-based therapies, banked iPSCs can serve as an effective tool for other applications, including drug discovery and disease modeling (39–42). Although the establishment of iPSC banks has gained recognition, technical challenges remain, such as efficient cryopreservation and storage (43, 44). Researchers are making constant efforts to develop optimal methods that robustly support the use of these cells in fundamental, preclinical and clinical research.

In addition to efficient cell reprograming, we demonstrated for the first time that urine-derived iPSCs from brain tumor patients differentiate into iMSCs. Previous research has established that iMSCs possess remarkable advantages over MSCs derived from organs or tissues (27). Among these advantages, iMSCs originate from an infinite cell source (i.e. iPSCs), thus overcoming the availability limitation for the manufacturing process (45). Moreover, while tissue-derived MSCs possess high heterogeneity that may interfere with their therapeutic effects (25), iMSCs are theoretically more homogeneous because they can generate from a single iPSC clone (26, 27). Consequently, iMSCs may yield more consistent and reproducible results, even when different batches of iMSCs are used. In this context, several reports have demonstrated that iMSCs, including urine-derived iMSCs, show increased therapeutic efficacy in experimental models of disease (3–8, 46). For instance, intra-myocardial administration of iMSCs provided better regenerative effects compared with bone marrow MSCs in a rodent model of myocardial infarction (6). Similarly, the intracranial delivery of iMSCs promoted more robust neuroprotective effects than umbilical cord-derived MSCs in a hypoxic–ischemic rat model (5). A comparative study showed that iMSCs differentiated from the urine of a healthy volunteer exhibited superior wound-healing properties than umbilical cord MSCs (8). Interestingly, iMSCs have been suggested to be safer than bone marrow-derived MSCs in cancer treatment, since they are less prone to promote epithelial–mesenchymal transition, invasion, stemness and growth of cancer cells (47). In a separated study, iMSCs obtained from aged individuals acquired a rejuvenation-associated gene signature, which may be associated with a greater proliferation and differentiation capacity than native MSCs (48). In our study, we demonstrated that iMSCs possess immunomodulatory capacity, supporting their therapeutic application for the treatment of inflammatory processes, such as those induced by radiotherapy (18, 49). This opens new avenues for the generation of more efficient MSC-based therapies in pediatric regenerative medicine. As a major limitation, our study was performed with a reduced number of samples, which prevented from conducting a robust comparative study. Further research will help to elucidate whether the therapeutic properties of BT-iMSCs are equivalent to those of nonT-iMSCs, or even to tissue derived MSCs.

In conclusion, iPSC-based therapies are making steady progress, with more than 100 ongoing or completed clinical trials for several diseases, including neurological diseases, cardiomyopathy or cancer. Interestingly, 40% of these studies include the participation of children, which evidence the value of this sophisticated iPSC technology in the field of pediatric research. Despite the booming advancement on iPSC-derived products, such as iMSCs, a number of hurdles still have to be overcome to exploit their clinical application (50). Among their limitations, safety is the main concern when transplanting iPSC-derived cells, since any residual iPSCs may result in the formation of teratomas (51). For this reason, the development of efficient methods for iPSC differentiation is a crucial step prior to application, in both preclinical and clinical studies. Our research represents a step forward in the development of patient-specific products based on iPSC systems for the treatment of several diseases, which could be applied not only in pediatric patients, but also in adults.

## Data availability statement

The original contributions presented in the study are included in the article/**Supplementary Materials**, further inquiries can be directed to the corresponding author/s.

## Ethics statement

The studies involving human participants were reviewed and approved by the Institutional Review Board (or Ethics Committee) of “Secretaría General de Investigación, Desarrollo e Innovación en Salud of Junta de Andalucia (protocol code PR-03-2020, approved on March 2nd 2021)” and the “Consejo Interministerial de Organismos Modificados Genéticamente (protocol code A/ES/21/03, approved on March 2nd 2021)”. Written informed consent to participate in this study was provided by the participants’ legal guardian/next of kin.

## Author contributions

CB-S, YA, JMS-P and VC-G contributed to conception and design of the study. CM-V and RC-L collected human samples and provided magnetic resonances images of patients. CB-S, YA, NA, JMS-P, LO-M and CP-M performed the experiments. CB-S, YA, JMS-P and VC-G analyzed the data. VC-G and CB-S wrote the manuscript and prepared figures. JMS-P and AM-M contributed to data interpretation and wrote sections of the manuscript. All authors contributed to manuscript revision, read, and approved the submitted version.
