# Supplementary material for: Generation of mesenchymal stromal cells from urine-derived iPSCs of pediatric brain tumor patients
Source: Front Immunol. 2023 Jan 26;14:1022676. doi: 10.3389/fimmu.2023.1022676 (PMC9910217; doi:10.3389/fimmu.2023.1022676)
Supplement: Supplementary file 1 [file DataSheet_1.pdf]

frontiers | Frontiers in Immunology

Generation of mesenchymal stromal cells from urine-derived iPSCs of pediatric brain tumor patients

Supplemental data

FIGURES AND TABLES

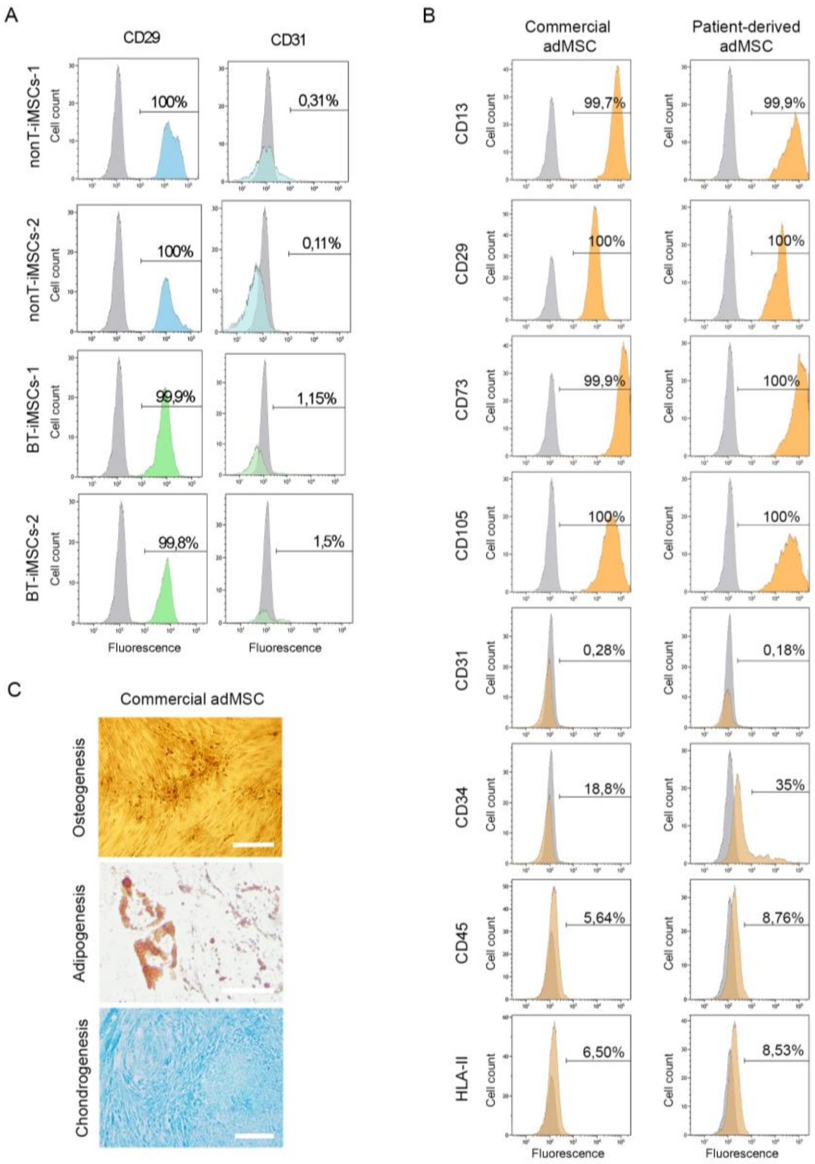

Supplemental Figure 1. Phenotypic characterization of MSCs by flow cytometry. (A) Flow cytometry analysis for CD29 and CD31 markers in iMSCs from BT and nonT patients. Note that cells were positive for CD29 while they were negative for CD31. (**B**) Flow cytometry analysis of a commercial adipose tissue-derived mesenchymal stem cell (adMSC) line and an adMSC line derived from a pediatric patient. Graphs show that cells express the MSC-specific markers CD13, CD29, CD73 and CD105, whereas they were negative for CD31, CD34, CD45, and HLA-II. (**C**) Images of the commercial adMSC line differentiated into osteocytes (scale bar 100µm), adipocytes (scale bar 50µm) and chondrocytes (scale bar 100µm), showing positive staining for Alizarin Red, Oil Red O and Alcian Blue respectively. Note that all these experiments were run at the same time than those presented in Figure 6C and Figure 6D.

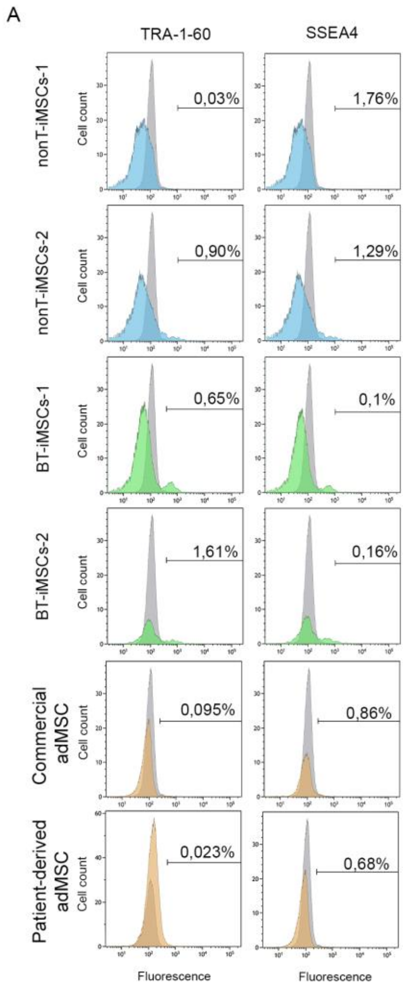

Supplemental Figure 2. Pluripotency markers in differentiated iMSCs. (A) Flow cytometry analysis showing the lack of expression of the pluripotency markers TRA-1-60 and SSEA4 in the generated iMSCs, in commercial adipose tissue-derived mesenchymal stem cells (adMSC) and in patient-derived adMSCs.

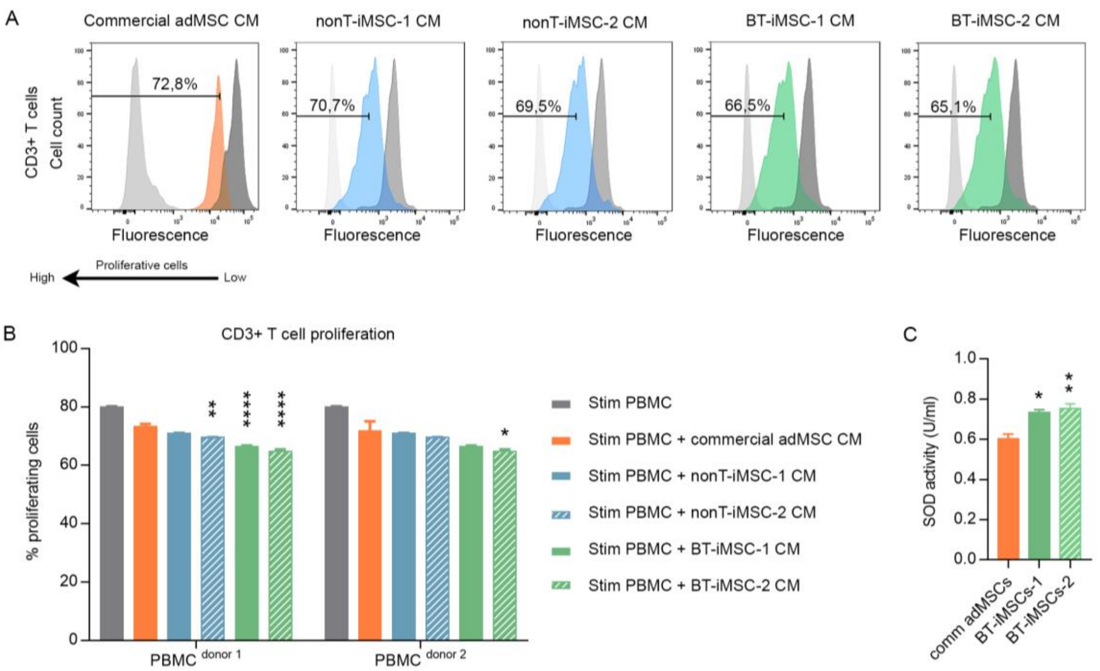

Supplemental Figure 3. Immunomodulatory capacity of MSC. (A) Representative flow cytometry plots showing the effect of conditioned media from commercial adipose tissue-derived mesenchymal stem cells (adMSC) or iMSCs on the proliferation of CD3+ T-cells. Stimulated PBMCs were exposed to the secretome for 72 hours. (**B**) Flow cytometry bar graph showing the percentage of proliferative $CD3^+$ T cells in response to the secretome of commercial adMSC and iMSCs. Stimulated PBMCs were exposed to the secretome for 72 hours. *p<0.01; **p<0.001; ****p<0.0001, compared to commercial adMSC. One-way ANOVA. (**C**) Bar graph showing the antioxidant actions of the secretome of commercial adMSCs and iMSCs measured by SOD activity. *p<0.05; **p<0.01; compared to commercial adMSC. One-way ANOVA. Data are represented as mean ± SEM.

Supplemental Table 1. List of primers.

Primer	Sequence (5' to 3')
BRACH	F: TGCTTCCCTGAGACCCAGTT<br>R: GATCACTTCTTTCCTTTGCAT
C-MYC	F: TAACTGACTAGCAGGCTTGTCG<br>R: TCCACATACAGTCCTGGATGATGATG
COX2	F: TAAGTGCGATTGTACCCGGAC<br>R: TTTGTAGCCATAGTCAGCATTGT
CXCR4	F: CACCGCATCTGGAGAACCA<br>R: GCCCATTTCCTCGGTGTAGTT
FOXA2	F: TTGCTGGTCGTTTGTTGTGGCT<br>R: TTCATGTTGCTCACGGAGGAGT
IDO	F: GCCCTTCAAGTGTTTCACCAA<br>R: CCAGCCAGACAAATATATGCGA
IL-1β	F: ATGATGGCTTATTACAGTGGCAA<br>R: GTCGGAGATTCGTAGCTGGA
IL-6	F: ACTCACCTCTTCAGAACGAATTG<br>R: CCATCTTTGGAAGGTTCAGGTTG
KLF4	F: TTCCTGCATGCCAGAGGAGCCC<br>R: AATGTATCGAAGGTGCTCAA
KOS	F: ATGCACCGCTACGACGTGAGCGC<br>R: ACCTTGACAATCCTGATGTGG
NANOG	F: GCAGAAGGCCTCAGCACCTA<br>R: AGGTTCCCAGTCGGGTTCA
NGN3	F: CTAAGAGCGAGTTGGCACTGA<br>R: GAGGTTGTGCATTCGATTGCG
OCT3/4	F: GCTGGAGAAGGATGTGGTCC<br>R: CGTTGTGCATAGTCGCTGCT
SEV	F: GGATCACTAGGTGATATCGAGC<br>R: ACCAGACAAGAGTTTAAGAGATATGTATC
SOX17	F: TGGACCGCACGGAATTTGAACA<br>R: TGTGTAACACTGCTTCTGGCCT
SOX2	F: CACTGCCCCTCTCACACATG<br>R: TCCCATTTCCCTCGTTTTTCT
TBP	Accesion: NM_003194*
TERT	F: ACATGGAGAACAAGCTGTT<br>R: TGTCGAGTCAGCTTGAGCA
TGF-β	F: CTAATGGTGGAAACCCACAACG<br>R: TATCGCCAGGAATTGTTGCTG
TNF-α	F: CCTCTCTCTAATCAGCCCTCTG<br>R: GAGGACCTGGGAGTAGATGAG
β-ACTIN	F: CGCACCACTGGCATTGTCAT<br>R: TTCTCCTTGATGTCACGCAC

* Supplied by Tataa Biocenter's Human Reference Gene Panel

Supplemental Table 2. List of primary antibodies.

Antibody	Manufacturer	Cat number	Dilution	Specificity
**AFP**	R&D System (Lille, France)	MAB1368	1:100	Endoderm
**CD3**	BD Biosciences (San Jose, CA, USA)	555333	1:20	T cell characterization
**CD4**	BD Biosciences (San Jose, CA, USA)	555346	1:20	T cell characterization
**CD8**	BD Biosciences (San Jose, CA, USA)	555366	1:20	T cell characterization
**CD13**	BD Biosciences (San Jose, CA, USA)	347406	1:40	MSC characterization
**CD14**	BD Biosciences (San Jose, CA, USA)	345784	1:20	MSC characterization
**CD29**	BD Biosciences (San Jose, CA, USA)	555443	1:40	MSC characterization
**CD31**	BD Biosciences (San Jose, CA, USA)	555445	1:20	MSC characterization
**CD34**	BD Biosciences (San Jose, CA, USA)	555822	1:40	MSC characterization
**CD45**	BD Biosciences (San Jose, CA, USA)	345808	1:40	MSC characterization
**CD73**	BD Biosciences (San Jose, CA, USA)	550257	1:100	MSC characterization
**CD90**	BD Biosciences (San Jose, CA, USA)	555595	1:100	MSC characterization
**CD105**	BD Biosciences (San Jose, CA, USA)	560839	1:40	MSC characterization
**HLA II**	BD Biosciences (San Jose, CA, USA)	555558	1:20	MSC characterization
**Nestin**	Abcam (Cambridge, United Kingdom)	ab6320	1:200	Ectoderm
**OCT3/4**	R&D System (Lille, France)	MAB1759	1:50	Pluripotency
**SMA**	Sigma-Aldrich (St. Louis, MO)	A5228	1:50	Mesoderm
**SSEA4**	Merck KGaA (Darmstadt, Germany)	90231	1:100	Pluripotency (ICC)
**SSEA4**	BD Biosciences (San Jose, CA, USA)	560128	1:5	Pluripotency (FC)
**TRA 1-60**	Merck KGaA (Darmstadt, Germany)	90232	1:100	Pluripotency (ICC)
**TRA 1-60**	BD Biosciences (San Jose, CA, USA)	560380	1:5	Pluripotency (FC)
**TRA 1-81**	Merck KGaA (Darmstadt, Germany)	90233	1:100	Pluripotency

Supplemental Table 3. Genomic alterations in the iPSCs from brain tumor pediatric patients.

Sample	Type	Chr	Start (bp)	End (bp)	Size (kbp)	OMIM ® Genes	OMIM ® Phenotype Loci
BT-iPSCs 1	LOH	3	q21.3	q22.1	3145937	LINC01565 (618259), RPN1 (180470), RAB7A (602298), ACAD9 (611103), GP9 (173515), ISY1 (612764), CNBP (116955), COPG1 (615525), HMCES (618288), H1-10 (602785), MBD4 (603574), IFT122 (606045), RHO (180380), PLXND1 (604282), TMCC1 (616242), TRH (613879), COL6A4P2 (616612), COL6A5 (611916), COL6A6 (616613), PIK3R4 (602610), ATP2C1 (604384), NEK11 (609779), NUDT16 (617381), MRPL3 (607118), CPNE4 (604208)	[Birth weight QTL 3] (613460), [Fasting plasma glucose level QTL 6] (613460), {Dermatitis, atopic, susceptibility to, 1} (603165), {Psoriasis susceptibility 5} (604316), Facial paresis, hereditary congenital, 1 (601471), Glaucoma 1C, primary open angle (601682), Deafness, autosomal dominant 18 (606012), Parkinson disease 21 (616361), Senior-Loken syndrome 3 (606995), Dandy-Walker syndrome (220200), Otosclerosis 5 (608787), {Alzheimer disease-15} (611155)
	Loss	3	p26.3	p26.1	6709543	CHL1 (607416), CNTN6 (607220), CNTN4 (607280), IL5RA (147851), TRNT1 (612907), CRBN (609262), LRRN1 (619623), SETMAR (609834), SUMF1 (607939), ITPR1 (147265), EGOT (611662), BHLHE40 (604256), ARL8B (616596), EDEM1 (607673)	{Inflammatory bowel disease 9} (608448), {Prostate cancer, hereditary, 5} (609299), {Stature QTL 5} (608982), 3p- syndrome (613792), Moyamoya disease (252350), Creatinine clearance QTL (607135), {Age-related hearing impairment 2} (612976)
	Gain	4	q21.23	q22.3	10587054	NKX6-1 (602563), CDS1 (603548), WDFY3 (617485), ARHGAP24 (610586), MAPK10 (602897), PTPN13 (600267), SLC10A6 (613366), AFF1 (159557), KLHL8 (611967), HSD17B13 (612127), HSD17B11 (612831), NUDT9 (606022), SPARCL1 (606041), DSPP (125485), DMP1 (600980), IBSP (147563), MEPE (605912), SPP1 (166490), PKD2 (173910), ABCG2 (603756), PPM1K (611065), HERC6 (609249), HERC5 (608242), PIGY (610662), HERC3 (605200), NAP1L5 (612203), FAM13A-AS1 (613300), FAM13A (613299), TIGD2 (612973), GPRIN3 (611241), SNCA (163890), MMRN1 (601456), CCSER1 (618934), GRID2 (602368), ATOH1 (601461), SMARCAD1 (612761), HPGDS (602598), PDLIM5 (605904), BMPR1B (603248)	{Intelligence QTL1} (603783), Narcolepsy 2 (605841), {Macroglobulinemia, Waldenstrom, susceptibility to, 2} (610430), {Mental health wellness-2} (603664), {Psoriasis susceptibility 3} (601454), {Autoimmune disease, susceptibility to, 4} (609400), Epilepsy, familial temporal lobe, 3 (611630), Chromosome 4q21 deletion syndrome (613509), Parietal foramina 3 (609566), Orofacial cleft 4 (608371), {Dermatitis, atopic, susceptibility to, 8} (613518), [Musical aptitude QTL 1] (612343), Myopia 11 (609994)

Gain	5	q32	q32	1556598	PPP2R2B (604325), DPYSL3 (601168), JAKMIP2 (611197), SPINK1 (167790), SCGB3A2 (606531), SPINK5 (605010), SPINK6 (615868)	{Bone size QTL} (609657), Macular dystrophy, butterfly-shaped pigmentary, 2 (608970), Glaucoma 1, open angle, M (610535), {Schizophrenia} (181510), Deafness, autosomal dominant 52 (607683), {Diabetes mellitus, insulin-dependent, 18} (605598), {Psoriasis susceptibility 11} (612599), Eosinophilia, familial (131400), {Autoimmune thyroid disease, susceptibility to, 2} (608174), {Celiac disease, susceptibility to, 2} (609754), {Dermatitis, atopic, susceptibility to, 6} (605845), {Malaria, intensity of infection} (248310), {Schistosoma mansoni infection, susceptibility/resistance to} (181460), Telangiectasia, hereditary hemorrhagic, type 3 (601101)
LOH	6	q11.1	q12	3446945	KHDRBS2 (610487), LGSN (611470), PTP4A1 (601585), PHF3 (607789), EYS (612424)	{Cataract 28, age-related cortical, susceptibility to} (609026), Mental retardation, autosomal recessive 24 (614345), Chromosome 6q11-q14 deletion syndrome (613544), Vestibulopathy, familial (193007), {Attention deficit-hyperactivity disorder} (608905), Mental retardation, autosomal recessive 30 (614342), Cardiomyopathy, dilated, 1K (605582)
Gain	6	q16.1	q16.3	9514378	EPHA7 (602190), MANEA (612327), FUT9 (606865), UFL1 (613372), FHL5 (605126), GPR63 (606915), NDUFAF4 (611776), MMS22L (615614), (616328), POU3F2 (600494), FBXL4 (605654), COQ3 (605196), PNISR (616653), USP45 (618439), CCNC (123838), PRDM13 (616741), MCHR2 (606111), SIM1 (603128), ASCC3 (614217), GRIK2 (138244)	Vestibulopathy, familial (193007), Cardiomyopathy, dilated, 1K (605582), Otosclerosis 7 (611572), {Schizophrenia} (603175), Atrial fibrillation, familial, 2 (608988), Chorioretinal atrophy, progressive bifocal (616842), Macular dystrophy 1, North Carolina type (616842), Lymphatic malformation 2 (611944), Generalized epilepsy with febrile seizures plus, type 8 (613828)
Gain	7	q31.32	q31.32	470455	AASS (605113), FEZF1 (613301), CADPS2 (609978)	Silver-Russell syndrome 2 (618905), Spinocerebellar ataxia 18 (607458), Deafness, autosomal recessive 14 (603678), Deafness, autosomal recessive 17 (603010), {Autism, susceptibility to, 9} (611015), {Stature QTL 2} (606256)
Gain	14	q32.33	q32.33	51493	FAM30A (616623)	Hemifacial microsomia (164210), Kagami-Ogata syndrome (608149), Microphthalmia, isolated 1 (251600), Temple syndrome (616222), [Creatine kinase, brain type, ectopic expression of] (123270), {Coronary heart disease, susceptibility to, 4} (608318),

							{Gene expression, variation in, QTL} (608875), {Myeloproliferative neoplasms, familial, susceptibility to} (616604), ?Hyperimmunoglobulin G1 syndrome (144120), Agammaglobulinemia 1 (147020), IgG2 deficiency, selective (147110)
	LOH	15	q21.3	q21.3	3261663	UNC13C (614568), RSL24D1 (613262), RAB27A (603868), PIGBOS1 (618809), PIGB (604122), CCPG1 (611326), PIERCE2 (619669), DNAAF4 (608706), PYGO1 (606902), PRTG (613261), NEDD4 (602278), RFX7 (612660), MNS1 (610766), TCF12 (600480), CGNL1 (607856)	Cholestasis-lymphedema syndrome (214900), {Human coronavirus sensitivity} (122460), {Hypertension, essential, susceptibility to, 2} (604329), Polyposis syndrome, hereditary mixed 1 (601228), {Colorectal cancer, susceptibility to, 4} (601228), Dyserythropoietic anemia, congenital, type III (105600), Cataract 25 (605728)
	LOH	X	p11.22	p11.1	7749302	DGKK (300837), SHROOM4 (300579), BMP15 (300247), NUDT10 (300527), EZHIP (301036), NUDT11 (300528), GSPT2 (300418), MAGED1 (300224), MAGED4 (300702), MAGED4B (300765), XAGE2 (300416), XAGE1B (300289), XAGE1A (300742), SSX8P (300543), SSX7 (300542), SSX2 (300192), SPANXN5 (300668), XAGE3 (300740), GPR173 (300253), TSPYL2 (300564), KANTR (301019), KDM5C (314690), IQSEC2 (300522), SMC1A (300040), HSD17B10 (300256), HUWE1 (300697), MIR98 (300810), MIRLET7F2 (300721), PHF8 (300560), FAM120C (300741), WNK3 (300358), TSR2 (300945), FGD1 (300546), GNL3L (300873), MAGED2 (300470), SNORA11 (300662), TRO (300132), PFKFB1 (311790), APEX2 (300773), ALAS2 (301300), PAGE2 (300738), PAGE5 (301009), PAGE3 (300739), MAGEH1 (300548), FOXR2 (300949), RRAGB (300725), KLF8 (300286), UBQLN2 (300264), NBDY (300992), SPIN2B (300517), SPIN2A (300621), FAAH2 (300654), ZXDB (300236), ZXDA (300235)	Optic atrophy 2, X-linked (311050), ?Mental retardation, X-linked, syndromic 12 (309545), {Diabetes mellitus, insulin-dependent, X-linked} (300136), {Graves disease, susceptibility to, X-linked} (300351), Mental retardation, X-linked 20 (300047), Prieto syndrome (309610), Mental retardation,X-linked 45 (300498), Angio serpiginosum (300652), Mental retardation, X-linked 14 (300062), Mental retardation, X-linked 84 (300505), Mental retardation, X-linked syndromic 7 (300218), Chromosome Xp11.23-p11.22 duplication syndrome (300801), Mental retardation, X-linked 81 (300433), Xp11.22 microduplication syndrome (300705), {Hypospadias 4, X-linked, susceptibility to} (300856), {Prostate cancer, hereditary, X-linked 2} (300704)
BT-iPSCs 2	LOH	1	p33	p32.3	3690266	AGBL4 (616476), ELAVL4 (168360), DMRTA2 (614804), FAF1 (604460), CDKN2C (603369), RNF11 (612598), EPS15 (600051), OSBPL9 (606737), NRDC (602651), RAB3B (179510), TXNDC12 (609448), ZFYVE9 (603755), ORC1 (601902), PRPF38A	{Anorexia nervosa, susceptibility to, 1} (606788), {Psoriasis susceptibility 7} (605606), Macrostomia (613545), Ptosis, hereditary congenital, 1 (178300), ?Pain sensitivity QTL1 (618377), Orofacial cleft 13 (613857), [Bone mineral density QTL 14] (612728), Forsythe-Wakeling syndrome

					(617031), TUT4 (613692), GPX7 (615784)	(613606), {Parkinson disease 10} (606852), {Stature QTL 23} (613548)
LOH	3	p21.31	p21.31	3914767	LZTFL1 (606568), CCR9 (604738), FYCO1 (607182), CXCR6 (605163), XCR1 (600552), CCR1 (601159), CCR3 (601268), CCR2 (601267), CCR5 (601373), CCRL2 (608379), LTF (150210), RTP3 (607181), LRRC2 (607180), TDGF1 (187395), ALS2CL (612402), TMIE (607237), PRSS50 (607950), MYL3 (160790), PTH1R (168468), NBEAL2 (614169), SETD2 (612778), KIF9 (607910), PTPN23 (606584), SCAP (601510), ELP6 (615020), CSPG5 (606775), SMARCC1 (601732), DHX30 (616423), MAP4 (157132), CDC25A (116947), CAMP (600474), ZNF589 (616702), NME6 (608294), FBXW12 (609075), PLXNB1 (601053), CCDC51 (618585), TMA7 (615808), ATRIP (606605), TREX1 (606609), SHISA5 (607290), PFKFB4 (605320), UCN2 (605902), COL7A1 (120120), UQCRC1 (191328), SLC26A6 (610068), CELSR3 (604264), NCKIPSD (606671), IP6K2 (606992), PRKAR2A (176910), SLC25A20 (613698), ARIH2 (605615), P4HTM (614584), WDR6 (606031), DALRD3 (618904), NDUFAF3 (612911), MIR191 (615150), IMPDH2 (146691), QRICH1 (617387), QARS1 (603727), USP19 (614471), LAMB2 (150325), KLHDC8B (613169), IHO1 (619190), MIR4271 (617176), USP4 (603486), GPX1 (138320), RHOA (165390), TCTA (600690), AMT (238310), NICN1 (611516), DAG1 (128239), BSN (604020), APEH (102645), MST1 (142408), RNF123 (614472), AMIGO3 (615691), GMPPB (615320), IP6K1 (606991), UBA7 (191325)	Creatinine clearance QTL (607135), Trichilemmal cyst 1 (609649), {Asperger syndrome susceptibility 4} (609954), Small-cell cancer of lung (182280), {Inflammatory bowel disease 12} (612241), Cholangitis, primary sclerosing (613806), {Celiac disease, susceptibility to, 9} (612007), {Hirschsprung disease, susceptibility to, 6} (606874), [Mean platelet volume QTL2] (612574), Zygodactyly 1 (609815)
Gain	6	q16.1	q16.3	918398	EPHA7 (602190), MANEA (612327), FUT9 (606865), UFL1 (613372), FHL5 (605126), GPR63 (606915), NDUFAF4 (611776), MMS22L (615614), (616328), POU3F2 (600494), FBXL4 (605654),	Vestibulopathy, familial (193007), Cardiomyopathy, dilated, 1K (605582), Otosclerosis 7 (611572), {Schizophrenia} (603175), Atrial fibrillation, familial, 2 (608988), Chorioretinal atrophy, progressive bifocal

						COQ3 (605196), PNISR (616653), USP45 (618439), CCNC (123838), PRDM13 (616741), MCHR2 (606111), SIM1 (603128), ASCC3 (614217), GRIK2 (138244)	(616842), Macular dystrophy 1, North Carolina type (616842), Lymphatic malformation 2 (611944), Generalized epilepsy with febrile seizures plus, type 8 (613828)
	LOH	7	q11.22	q11.23	3133507	POM121 (615753), TRIM73 (612549), TRIM74 (612550), NSUN5 (615732), TRIM50 (612548), FKBP6 (604839), FZD9 (601766), BAZ1B (605681), BCL7B (605846), TBL2 (605842), MLXIPL (605678), VPS37D (610039), DNAJC30 (618202), BUD23 (615733), STX1A (186590), ABHD11-AS1 (612545), CLDN3 (602910), CLDN4 (602909), METTL27 (612546), TMEM270 (612547), ELN (130160), LIMK1 (601329), EIF4H (603431), MIR590 (615070), LAT2 (605719), RFC2 (600404), CLIP2 (603432), GTF2IRD1 (604318), GTF2I (601679), NCF1 (608512), GTF2IRD2 (608899), CASTOR2 (617033), GTF2IRD2B (608900), POM121C (615754), HIP1 (601767)	Silver-Russell syndrome 2 (618905), Cardiomyopathy, hypertrophic, 21 (614676), {Prostate cancer, susceptibility to, 4} (608658), Aneurysm, intracranial berry, 1 (105800), {Esophagitis, eosinophilic, 1} (610247), ?EEC syndrome-1 (129900), Chromosome 7q11.23 deletion syndrome, distal, 1.2Mb (613729), Chromosome 7q11.23 duplication syndrome (609757), Williams-Beuren syndrome (194050)
	LOH	7	q21.11	q21.13	4151647	GRM3 (601115), ELAPOR2 (614048), DMTF1 (608491), TMEM243 (616993), TP53TG1 (616403), CROT (606090), ABCB4 (171060), ABCB1 (171050), RUNDC3B (617295), SLC25A40 (610821), DBF4 (604281), ADAM22 (603709), SRI (182520), STEAP4 (611098)	Silver-Russell syndrome 2 (618905), Cardiomyopathy, hypertrophic, 21 (614676), {Prostate cancer, susceptibility to, 4} (608658), ?EEC syndrome-1 (129900), {Stature QTL 11} (612223), {Malignant hyperthermia susceptibility 3} (154276)
	Loss	8	q24.22	q24.3	7096185	CCN4 (603398), NDRG1 (605262), ST3GAL1 (607187), ZFAT (610931), MIR30B (619018), MIR30D (619019), KHDRBS3 (610421), COL22A1 (610026), KCNK9 (605874), TRAPPC9 (611966)	Chondrocalcinosis with early-onset osteoarthritis (600668), Fetal hemoglobin quantitative trait locus 4 (606789), Hashimoto thyroiditis (140300), Mungan syndrome (611376), Epilepsy, childhood absence, 1 (600131), [Bone mineral density QTL 10] (612113), [Bone size quantitative trait locus 3] (610649), {Colorectal cancer, susceptibility to, 2} (611469), {Epilepsy, idiopathic generalized, susceptibility to, 1} (600669), {Prostate cancer, hereditary, 10} (611100), {Age-related hearing impairment 1} (612448), Orofacial cleft 12 (612858)

Loss	10	q11.21	q11.23	8474866	ZNF33B (194522), BMS1 (611448), RET (164761), CSGALNACT2 (616616), RASGEF1A (614531), FXYD4 (616926), HNRNPF (601037), ZNF239 (601069), ZNF32 (194539), CXCL12 (600835), RASSF4 (610559), DEPP1 (611309), ZNF22 (194529), ALOX5 (152390), MARCHF8 (613335), WASHC2C (613631), TIMM23 (605034), NCOA4 (601984), MSMB (157145), NPY4R (601790), GPRIN2 (611240), SYT15 (608081), PTPN20 (610630), GDF10 (601361), GDF2 (605120), RBP3 (180290), ANXA8 (602396), FRMPD2 (613323), MAPK8 (601158), ARHGAP22 (610585), WDFY4 (613316), LRRC18 (619002), DRGX (606701), ERCC6 (609413), CHAT (118490), SLC18A3 (600336), OGDHL (617513), PARG (603501), ASAH2 (611202)	Prostate adenocarcinoma (601188), Deafness, autosomal recessive 33 (607239), Usher syndrome, type IK (614990), {Autoimmune thyroid disease, susceptibility to, 4} (608176), Tooth agenesis, selective, 5 (610926), Hypotrichosis 9 (614237)
LOH	10	q11.21	q11.22	3341585	RASSF4 (610559), DEPP1 (611309), ZNF22 (194529), ALOX5 (152390), MARCHF8 (613335), WASHC2C (613631), TIMM23 (605034), NCOA4 (601984), MSMB (157145), NPY4R (601790), GPRIN2 (611240), SYT15 (608081), PTPN20 (610630), GDF10 (601361), GDF2 (605120), RBP3 (180290), ANXA8 (602396)	Prostate adenocarcinoma (601188), Deafness, autosomal recessive 33 (607239), Usher syndrome, type IK (614990), {Autoimmune thyroid disease, susceptibility to, 4} (608176), Tooth agenesis, selective, 5 (610926)
Gain	14	q32.33	q32.33	682843	FAM30A (616623)	Hemifacial microsomia (164210), Kagami-Ogata syndrome (608149), Microphthalmia, isolated 1 (251600), Temple syndrome (616222), [Creatine kinase, brain type, ectopic expression of] (123270), {Coronary heart disease, susceptibility to, 4} (608318), {Gene expression, variation in, QTL} (608875), {Myeloproliferative neoplasms, familial, susceptibility to} (616604), ?Hyperimmunoglobulin G1 syndrome (144120), Agammaglobulinemia 1 (147020), IgG2 deficiency, selective (147110)
LOH	20	q11.1	q11.21	3077302	DEFB118 (607650), DEFB119 (615997), DEFB121 (616075), DEFB122 (616077), DEFB123 (616076), REM1 (610388), HM13 (607106), ID1 (600349), COX4I2 (607976), BCL2L1 (600039), TPX2 (605917), MYLK2 (606566), FOXS1 (602939), DUSP15 (616776), PDRG1 (610789),	Dystonia-17, primary torsion (612406), Mulchandani-Bhoj-Conlin syndrome (617352), Spinocerebellar ataxia, autosomal recessive 6 (608029), {Hypertension, essential, susceptibility to, 5} (610261), {Melanoma, cutaneous malignant, 7} (612263)

						HCK (142370), TM9SF4 (617727), PLAGL2 (604866), POFUT1 (607491), KIF3B (603754), ASXL1 (612990), NOL4L (618893), COMMD7 (616703), DNMT3B (602900), MAPRE1 (603108), SUN5 (613942), BPIFB2 (614108), BPIFB6 (614110), BPIFB3 (615717), BPIFB4 (615718), BPIFA4P (607627), BPIFA1 (607412)	
	Gain	X	p22.33	p22.31	662228	DHRSX (301034), ZBED1 (300178), CD99 (313470), CD99 (450000), XG (300879), GYG2 (300198), ARSD (300002), ARSL (300180), ARSH (300586), ARSF (300003), MXRA5 (300938), PRKX (300083), NLGN4X (300427), VCX3A (300533), PUDP (306480), STS (300747), VCX (300229), PNPLA4 (300102), VCX2 (300532), VCX3B (300981), ANOS1 (300836), FAM9A (300477), FAM9B (300478)	Turner syndrome-associated neurocognitive phenotype (313000), [Visuospatial/perceptual abilities] (313000), {Hodgkin disease susceptibility, pseudoautosomal} (300221), Corneal dystrophy, Lisch epithelial (300778), Episodic muscle weakness, X-linked (300211), FG syndrome 3 (300406), Mental retardation, X-linked 2 (300428), Ocular albinism with sensorineural deafness (300650), Aicardi syndrome (304050), Aneurysm, intracranial berry, 5 (300870), Goiter, multinodular, 2 (300273), [XG blood group system, Xg(a-) phenotype (314705)
	LOH	X	p11.22	p11.1	5173184	GPR173 (300253), TSPYL2 (300564), KANTR (301019), KDM5C (314690), IQSEC2 (300522), SMC1A (300040), HSD17B10 (300256), HUWE1 (300697), MIR98 (300810), MIRLET7F2 (300721), PHF8 (300560), FAM120C (300741), WNK3 (300358), TSR2 (300945), FGD1 (300546), GNL3L (300873), MAGED2 (300470), SNORA11 (300662), TRO (300132), PFKFB1 (311790), APEX2 (300773), ALAS2 (301300), PAGE2 (300738), PAGE5 (301009), PAGE3 (300739), MAGEH1 (300548), FOXR2 (300949), RRAGB (300725), KLF8 (300286), UBQLN2 (300264), NBDY (300992), SPIN2B (300517), SPIN2A (300621), FAAH2 (300654), ZXDB (300236), ZXDA (300235)	Optic atrophy 2, X-linked (311050), ?Mental retardation, X-linked, syndromic 12 (309545), {Diabetes mellitus, insulin-dependent, X-linked} (300136), {Graves disease, susceptibility to, X-linked} (300351), Mental retardation, X-linked 20 (300047), Prieto syndrome (309610), Mental retardation,X-linked 45 (300498), Angio serpiginosum (300652), Mental retardation, X-linked 14 (300062), Mental retardation, X-linked 84 (300505), Mental retardation, X-linked syndromic 7 (300218), Chromosome Xp11.23-p11.22 duplication syndrome (300801), Mental retardation, X-linked 81 (300433), Xp11.22 microduplication syndrome (300705), {Hypospadias 4, X-linked, susceptibility to} (300856), {Prostate cancer, hereditary, X-linked 2} (300704)

	LOH	X	q25	q26.1	3476699	ACTRT1 (300487), SMARCA1 (300012), OCRL (300535), APLN (300297), XPNPEP2 (300145), SASH3 (300441), ZDHHC9 (300646), UTP14A (300508), BCORL1 (300688), ELF4 (300775), AIFM1 (300169)	[Social cognition] (300082), {Migraine, familial typical, susceptibility to, 2} (300125), {Mycobacterium tuberculosis, susceptibility, X-linked} (300259), {Parkinson disease 12} (300557), ?Amelogenesis imperfecta, type IE, X-linked 2 (301201), Mental retardation, X-linked 53 (300324), {Coronary heart disease, susceptibility to, 3} (300464), Myopia 13 (300613), Mental retardation, X-linked 82 (300518), Radial ray deficiency (300378), Spastic paraplegia 34, X-linked (300750), {Stature QTL 6} (300591), Albinism-deafness syndrome (300700), Ptosis, hereditary congenital 2 (300245), Bazex syndrome (301845), Dermoids of cornea (304730), Corneal dystrophy, endothelial, X-linked (300779), Xq25 duplication syndrome (300979), Thoracoabdominal syndrome (313850), Mental retardation, X-linked 46 (300436), X inactivation, familial skewed, 2 (300179), Spinocerebellar ataxia, X-linked 5 (300703), Charcot-Marie-Tooth neuropathy, X-linked recessive, 3 (302802), Gustavson syndrome (309555), Mental retardation, X-linked 42 (300372), Split hand/foot malformation 2 (313350), ?Craniofacioskeletal syndrome (300712), Retinitis pigmentosa 24 (300155), Woods-Black-Norbury syndrome (300076)
	LOH	X	q26.2	q26.3	3045352	MIR19B2 (300722), MIR20B (300950), MIR106A (300792), PHF6 (300414), HPRT1 (308000), MIR503 (300865), MIR424 (300682), PLAC1 (300296), MOSPD1 (300674), RTL8C (300213), ZNF75D (314997), ZNF449 (300627), CT45A1 (300648), CT45A3 (300794), CT45A5 (300796), CT45A6 (300797), CT45A2 (300793), SAGE1 (300359), SLC9A6 (300231), FHL1 (300163), MAP7D3 (300930), BRS3 (300107), HTATSF1 (300346), VGLL1 (300583), CD40LG (300386), ARHGEF6 (300267), RBMX (300199), GPR101 (300393)	[Social cognition] (300082), {Migraine, familial typical, susceptibility to, 2} (300125), {Mycobacterium tuberculosis, susceptibility, X-linked} (300259), ?Amelogenesis imperfecta, type IE, X-linked 2 (301201), Mental retardation, X-linked 53 (300324), {Coronary heart disease, susceptibility to, 3} (300464), Myopia 13 (300613), Albinism-deafness syndrome (300700), Ptosis, hereditary congenital 2 (300245), Bazex syndrome (301845), Dermoids of cornea (304730), Mental retardation, X-linked 46 (300436), X inactivation, familial skewed, 2 (300179), Spinocerebellar ataxia, X-linked 5 (300703), Charcot-Marie-Tooth neuropathy, X-linked recessive, 3 (302802), Gustavson syndrome (309555), Mental retardation, X-linked 42 (300372), Split

								hand/foot malformation 2 (313350), ?Craniofacioskeletal syndrome (300712), Retinitis pigmentosa 24 (300155), Woods-Black-Norbury syndrome (300076), 46XX sex reversal 3 (300833), Chromosome Xq26.3 duplication syndrome (300942)

Supplemental Table 4. Growth rate and doubling time of cultured iMSCs at passage 1.

	nonT-iMSCs-1	nonT-iMSCs-2	BT-iMSCs-1	BT-iMSCs-2
Growth rate (% per day)	3.75	4.5	11.22	12.39
Doubling Time (days)*	18.48	15.4	6.18	5.6

** Doubling time= Duration∗Ln(2)/Ln(Final concentration/Initial concentration)*
